# Association between pulse pressure, systolic blood pressure and the risk of rapid decline of kidney function among general population without hypertension: results from the China health and retirement longitudinal study (CHARLS)

**DOI:** 10.1186/s12967-021-03176-8

**Published:** 2021-12-20

**Authors:** Huai-yu Wang, Qinqin Meng, Chao Yang, Yafeng Wang, Guilan Kong, Yaohui Zhao, Fang Wang, Luxia Zhang

**Affiliations:** 1 National Institute of Health Data Science, Peking University, 38 Xueyuan Road, Haidian District, Beijing, 100191 China; 2 School of Public Health, Peking University, Beijing, China; 3 Institute of Social Science Survey, Peking University, Beijing, China; 4 Renal Division, Department of Medicine, Peking University First Hospital, 8 Xishiku Street, Xicheng District, Beijing, 100034 China; 5 National School of Development, Peking University, Beijing, China

**Keywords:** Blood pressure, Chronic kidney disease, Risk indicator, Middle and old aged general population

## Abstract

**Background:**

Association between blood pressure (BP) and kidney function among the middle and old aged general population without hypertension remains unclear.

**Methods:**

Participants aged ≥ 45 years, with complete data in 2011 and 2015 interviews of the China Health and Retirement Longitudinal Study(CHARLS), and without pre-existing hypertension were included. Systolic BP (SBP) was categorized as low (< 120 mmHg), medium (120–129 mmHg), and high (120–139 mmHg). Diastolic BP (DBP) was categorized as low (< 60 mmHg), medium (60–74 mmHg), and high (75–89 mmHg). Pulse pressure (PP) was categorized as normal (< 60 mmHg) and high (≥ 60 mmHg). The outcome was defined as rapid decline of estimated glomerular filtration rate(eGFR, decline ≥ 4 ml/min/1.73 m^2^/year). BP combination was designed according to the category of SBP and PP. The association between BP components, types of BP combination, and the risk of rapid decline of eGFR was analyzed using multivariate logistic regression models, respectively. Age-stratified analyses were conducted.

**Results:**

Of 4,534 participants included, 695(15.3%) individuals were recognized as having rapid decline of eGFR. High PP[odds ratio(OR) = 1.34, 95%confidence interval(CI) 1.02–1.75], low SBP (OR = 1.28, 95%CI 1.03–1.59), and high SBP (OR = 1.32, 95% CI 1.02–1.71) were significantly associated with the risk of eGFR decline. Low SBP were associated with 65% increment of the risk of eGFR decline among participants aged < 55 years. The combination of high SBP and high PP (OR = 1.79, 95% CI 1.27–2.54) and the combination of low SBP and high PP (OR = 3.07, 95% CI 1.24–7.58) were associated with the increased risk of eGFR decline among the middle and old aged general population.

**Conclusion:**

Single and combination of high PP and high SBP could be the risk indicators of eGFR decline among the middle and old aged general population.

**Supplementary Information:**

The online version contains supplementary material available at 10.1186/s12967-021-03176-8.

## Background

Chronic kidney disease (CKD) is one of the global public health challenges due to the increasing prevalence, multiple morbidities, and the heavy burden on healthcare system [1–3]. Compared with the high prevalence (around 11%) [4, 5], the awareness of CKD was only 10% [5, 6]. Less than 5% of patients in the early stages of CKD were aware of their disease [7]. Subject to the asymptomatic feature of CKD in early stages, risk recognition and monitor are essential for the early prevention of CKD. As to the population having comorbidities related to CKD, such as hypertension, the control of the comorbidity is the modifiable risk indicator for the decline of kidney function. [8] As to the general population without CKD-related comorbidities, the middle and old aged individuals are generally considered as the target population for risk monitor of CKD since the process of ageing results in the change of structure and function of the kidney [8, 9]. However, the strategy of risk monitor of the general population remains to be explored. The current guidelines on CKD and blood pressure (BP) recommended the routine assessment of eGFR and albuminuria among all patients with hypertension [10–12]. Nevertheless, the presence of eGFR decline and albuminuria means the incidence of CKD and emphasizes the timely treatment of CKD, rather than the early prevention and risk evaluation of CKD. An effective risk indicator for the early risk-recognition of decline in kidney function among the middle and old aged general population is urgently needed. With consideration of the feature of general population who are less tending to receive invasive and costly examination in clinics, an easily accessible, pragmatic and cost-saving approach would be feasible for the risk recognition and monitor among the middle and old aged general population.

There is no doubt that BP is strongly associated with CKD and hypertension, which are cause and consequence to each other [12]. The anti-hypertensive treatment is recommended to preserve the kidney function among patients with CKD [12], and the lower levels of systolic BP (SBP) is associated with the reduced risk of incident CKD among patients with hypertension [13]. Prevention of hypertension is a widely accepted approach to prevent the risk of CKD among the general population without pre-existing hypertension. However, the goal of SBP for different outcomes in different population has been ongoing debate for decades [14]. The BP < 130/80 mmHg was acknowledged as the goal of BP control among patients with hypertension and CKD [10, 12], while the BP goal for kidney function preservation among the general population without hypertension remains under-investigated.

Pulse pressure (PP) is an important BP component, which is calculated by the difference of SBP and diastolic BP (DBP), determined by the arterial and cardiac function, and often used as a surrogate indicator of arterial stiffness [15, 16]. With accumulating evidence, the prognostic significance of PP on cardiovascular disease (CVD) and mortality, especially among the middle and old aged population, were increasingly considered [10] The 2018 European guidelines for arterial hypertension recommended the routine measurement of PP (Class IIb, Level C) [10]. Compared with SBP and DBP, PP showed a stronger association with the cardiac risk among the population with or without CKD [17–23]. Additionally, independent association distinguishing from that of SBP and DBP was reported between PP and cardiovascular disease (CVD), which indicated the potentially unique pathogenesis between PP and CVD [24]. However, regards to the prognostic association of PP and incident CKD, previous studies showed inconsistent results [25–29]. The heterogeneity of the study population and design might lead to the inconsistency since genetic researches demonstrated the substantially ethnic-specific association between genes and BP components [30–32, 32]. Additionally, the improved prognostic significance of the combination of PP and other BP components was observed on predicting the risk of CVD and cardiac mortality among the middle and old aged population [33, 34]. Given the shared pathogenesis of CKD and CVD such as hemodynamic stress and vascular stiffness [35], and considering the easily-accessible and cost-saving feature of BP components, it is worth investigating the association between PP as well as the combination of BP components and the change of kidney function among the middle and old aged general population.

Hence, the present study investigated the association between BP components and the risk of rapid decline of kidney function among the middle and old aged general population without hypertension based on a national cohort study in China. The association between combination of BP components and the risk of rapid decline of kidney function was evaluated. Given the strong linkage between ageing and the levels of BP, age-stratified analyses were also performed.

## Materials and methods

### Population

The present study is based on the China Health and Retirement Longitudinal Study (CHARLS), which is a nationally representative survey of the Chinese population aged 45 years or older and their spouses [36]. A design of four-stage, stratified, cluster sampling was applied. The baseline of CHARLS was conducted between 2011 and 2012 [37]. The blood samples were collected in 2011 and 2015 through a standardized process which was detailly described elsewhere (http://charls.pku.edu.cn/index/en.html).

**Fig. 1:**
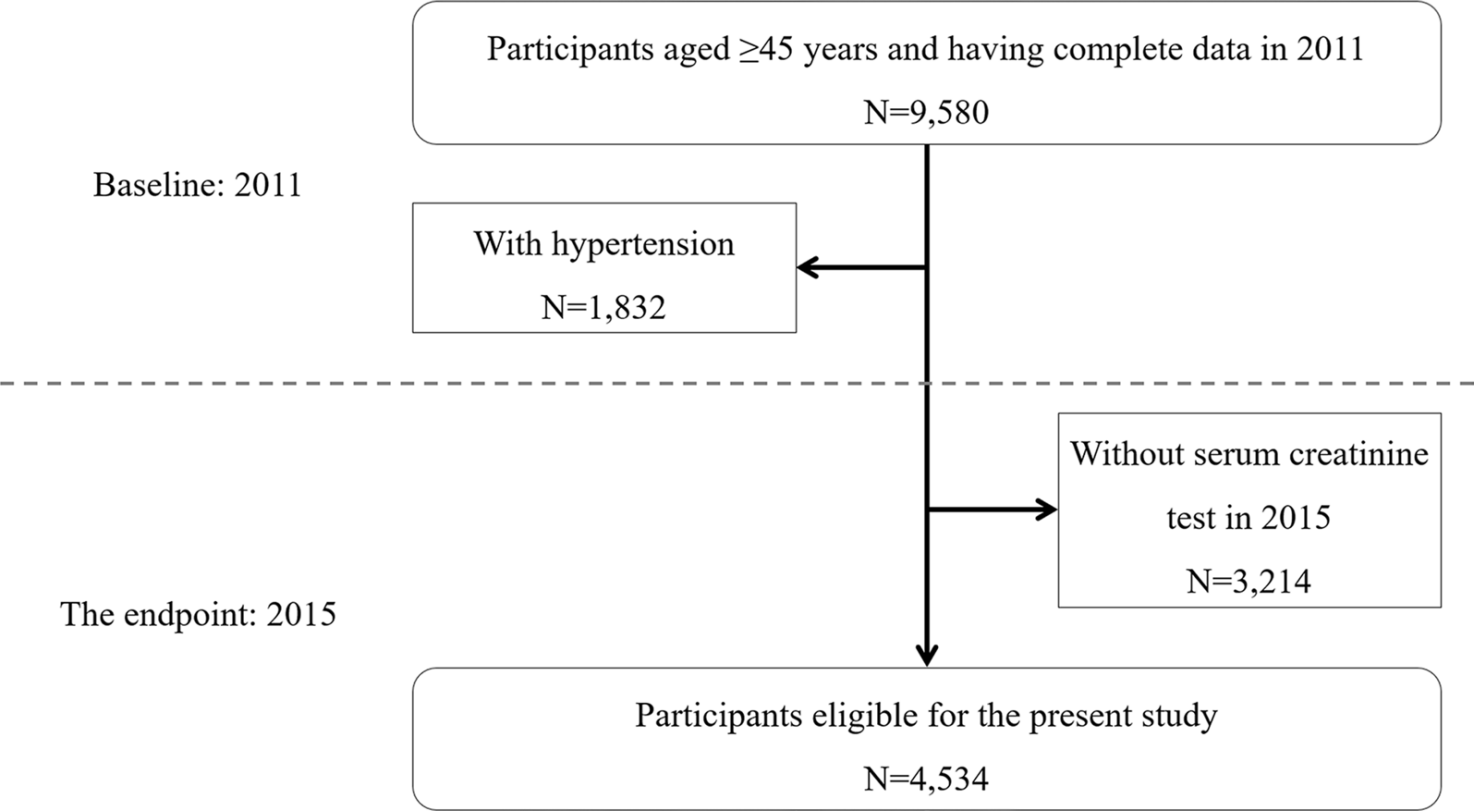
Flow chart for participants selection

A total of 9580 participants aged ≥ 45 years and completed the questionnaires, physical examination and blood sample collection in 2011 were included. A total of 1,832 participants diagnosed as hypertension, showing SBP ≥ 140 mmHg, or showing DBP ≥ 90 mmHg were excluded. Another 3,214 participants were excluded due to the absence of serum creatinine (SCr) tests in 2015. Ultimately, 4,534 participates were eligible for the present study. (Fig. 1).

This study was approved by the Biomedical Ethics Review Committee of Peking University. All participants gave written informed consent before participation.

### Data collection and specimen

The demographic, socio-economic, and health status were collected by structured questionnaires with a face-to-face computer-assisted personal interview [36]. The blood samples were stored at − 70℃, and measured in a central laboratory in Beijing [37]. Serum creatinine (SCr, mg/dL) was measured by the rate-blanked and compensated Jaffe creatinine method. Glycosylated hemoglobin (HbA1c, %) was measured by boronate affinity HPLC. Low-density lipoprotein (LDL, mg/dL) were measured by enzymatic colormetric test. Uric acid (UA, mg/dL) were measured by uricase and catalase.

### Variables

Demographic and clinical data in 2011 was defined as the information at baseline. Age, gender, and the status of smoke and drink (never, past, current) were recorded. Body mass index (BMI, kg/m^2^) was calculated as weight divided by height squared. Diabetes were defined by self-report history of diabetes, use of hypoglycaemic agents, or a fasting plasma glucose ≥ 7.0 mmol/L. Blood pressure was measured three times (with 45 s apart) using a HEM-7200 electronic monitor (Omron (Dalian) Co., LTD., Dalian, China). The average of three readings was calculated. PP was defined as the differential of SBP and DBP.

According to 2018 ESC/ESH Guidelines for the management of arterial hypertension, PP ≥ 60 mmHg was the risk factor of asymptomatic hypertension-mediated organ damage in older adults [10]. Therefore, the present study defined PP category as normal PP (< 60 mmHg) and high PP (≥ 60 mmHg). As to the target range of SBP, the 2018 ESC/ESH Guidelines recommended 130–139 mmHg for most patients and 120–129 mmHg for some patients to enhance the cerebrovascular protection, but suggested to avoid SBP < 120 mmHg to balance the potential benefit and harm on hemodynamics. [10] Accordingly, the present study defined the SBP category as low SBP (< 120 mmHg), medium SBP (120–129 mmHg) and high SBP (130–139 mmHg). As to the category of DBP, the threshold of diastolic hypotension was adopted to define the group of low DBP (< 60 mmHg) [38]. The J-shape curve point of DBP among the population without cardiovascular disease reported by the Framingham study, which was 75 mmHg, was adopted to define the groups of medium DBP (60–74 mmHg) and high DBP (75–89 mmHg) [39].

The following combination of SBP and PP, named BP combination, was designed according to the category of SBP and PP: normal SBP and normal PP, high SBP or high PP, high SBP and high PP, low SBP and normal PP, low SBP and high PP.

### Outcomes

Kidney function was evaluated according to the levels of eGFR. Levels of eGFR in 2011 and in 2015 were defined as the kidney function at baseline and at the endpoint, respectively. The rapid decline of eGFR was defined as eGFR decline ≥ 4 ml/min/1.73m^2^/year. The eGFR was calculated by the Chronic Kidney Disease Epidemiology Collaboration (CKD-EPI) equation [40].

### Statistics

The demographic and clinical characteristics at baseline were compared among participants with the different levels of eGFR. Levels and changes of eGFR [in 2011 (continuous), in 2015 (continuous), annual decline of eGFR (continuous), rapid decline of eGFR (categorical)] were compared among participants in different categories of BP components. Methods of oneway ANOVA, Kruskal–Wallis tests and Chi-square tests were applied for normal distributed, skewed distributed and categorical variables, respectively.

Multivariate logistic regression was applied to examine the association between categories of BP component (PP category, SBP category and DBP category), strategies of BP combination (medium SBP and normal PP, high SBP or high PP, high SBP and high PP, low SBP and normal PP, low SBP and high PP) and the risk of rapid decline of eGFR, respectively. Covariates including age (continuous), gender (male vs. female), smoke (never vs. past vs. current), drink (never vs. past vs. current), BMI (continuous), HbA1c (continuous), LDL (continuous), UA (continuous) and eGFR at baseline (continuous) were adjusted. The results were presented as odds ratio (OR) with 95% confidence interval (CI). Age stratified analyses according to the groups of 45–54 years, 55–64 years, and ≥ 65 years were performed.

All *P* value were two-tailed and *P* value < 0.05 was considered to be statistical significance. All analyses were conducted using Stata version 16.0 (Stata Corp LP, College Station, TX, USA).

## Results

### Population characteristics

The population characteristics at baseline were presented in overall and the levels of eGFR. (Table 1) In total, 4534 participants with the mean age of 57.8 ± 8.4 years were included in the present study. Among them, 1313(39.0%) participants showed the levels of eGFR 60–89 ml/min/1.73m^2^ and 81(1.8%) participants had eGFR < 60 ml/min/1.73m^2^. In contrast to those with eGFR ≥ 90 ml/min/1.73m^2^, participants with lower eGFR were older and with higher proportions of former smoker and former drinkers. (*P* < 0.05) More participants with eGFR < 60 ml/min/1.73m^2^ had diabetes. (eGFR ≥ 90 ml/min/1.73m^2^ 13.1% vs. eGFR < 60 ml/min/1.73m^2^ 27.1%; *P* = 0.003) Participants with decreased eGFR showed significantly lower levels of LDL, but higher levels of UA. (*P* < 0.01).

**Table 1:** Comparison of demographic and clinical characteristics among participants with different levels of eGFR at baseline

Characteristics	Overall	eGFR (ml/min/1.73m^2^)			*P* value
		≥ 90	60–89	< 60	
In Total (n, %)	4534(100.0)	3140(69.2)	1313(39.0)	81(1.8)	
Age (years, mean ± SD)	57.8 ± 8.4	55.9 ± 7.5	61.7 ± 8.6	68.9 ± 8.4	< 0.001
Gender (n, %)					0.530
Male	2092(46.1)	1439(45.8)	611(46.5)	42(51.9)	
Female	2442(53.9)	1701(51.2)	702(53.5)	39(48.1)	
Smoke (n, %)					0.019
Never	2805(62.1)	1966(62.7)	792(60.7)	47(58.8)	
Past	387(8.6)	240(7.7)	136(10.4)	11(13.8)	
Current	1326(29.4)	928(29.6)	376(28.8)	22(27.5)	
Drink (n, %)					0.002
Never	2669(58.9)	1837(58.6)	777(59.2)	55(67.9)	
Past	349(7.7)	215(6.9)	126(9.6)	8(9.9)	
Current	1512(33.4)	1084(34.6)	410(31.2)	18(22.2)	
BMI (kg/m^2^, mean ± SD)	23.3 ± 3.7	23.3 ± 3.7	23.3 ± 3.8	22.5 ± 4.4	0.170
Diabetes (n, %)					0.003
No	3521(86.3)	2464(86.9)	1006(85.7)	51(72.9)	
Yes	559(13.7)	372(13.1)	168(14.3)	19(27.1)	
HbA1c (%, mean ± SD)	5.25 ± 0.77	5.25 ± 0.79	5.24 ± 0.72	5.37 ± 0.90	0.311
LDL (mg/dl, mean ± SD)	115.4 ± 33.7	113.5 ± 32.7	120.0 ± 35.8	113.6 ± 34.7	< 0.001
UA (mg/dl, mean ± SD)	4.3 ± 1.2	4.1 ± 1.1	4.8 ± 1.2	5.9 ± 1.6	< 0.001
PP (mmHg, mean ± SD)	48.1 ± 8.8	47.5 ± 8.6	49.3 ± 9.2	51.6 ± 10.2	< 0.001
PP category (n, %)					< 0.001
Normal: < 60 mmHg	4057(89.5)	2869(91.4)	1124(85.6)	64(79.0)	
High: ≥ 60 mmHg	477(10.5)	271(8.6)	189(14.4)	17(21.0)	
SBP (mmHg, mean ± SD)	118.6 ± 11.8	118.0 ± 11.6	119.7 ± 12.1	122.3 ± 11.2	< 0.001
SBP category (n, %)					< 0.001
Low: < 120 mmHg	2398(52.9)	1728(55.0)	644(49.1)	26(32.1)	
Medium: 120–129 mmHg	1187(26.2)	827(26.3)	327(24.9)	33(40.7)	
High: 130–139 mmHg	949(20.9)	585(18.6)	342(26.1)	22(27.2)	
DBP (mmHg, mean ± SD)	70.5 ± 8.9	70.5 ± 8.9	70.5 ± 9.1	70.7 ± 8.9	0.950
DBP category (n, %)					0.836
Low: < 60 mmHg	531(11.7)	364(11.6)	160(12.2)	7(8.6)	
Medium: 60–74 mmHg	2502(55.2)	1733(55.2)	725(55.2)	44(54.3)	
High: 75–89 mmHg	1501(33.1)	1043(33.2)	428(32.6)	30(37.0)	

*eGFR* estimated glomerular filtration, *SD* standard deviation, *IQR* interquartile range, *BMI* body mass index, *HbA1c* hemoglobin A1c, *LDL* low-density lipoprotein, *UA* uric acid, *PP* pulse pressure, *SBP* systolic pressure, *DBP* diastolic pressure

**Fig. 2:**
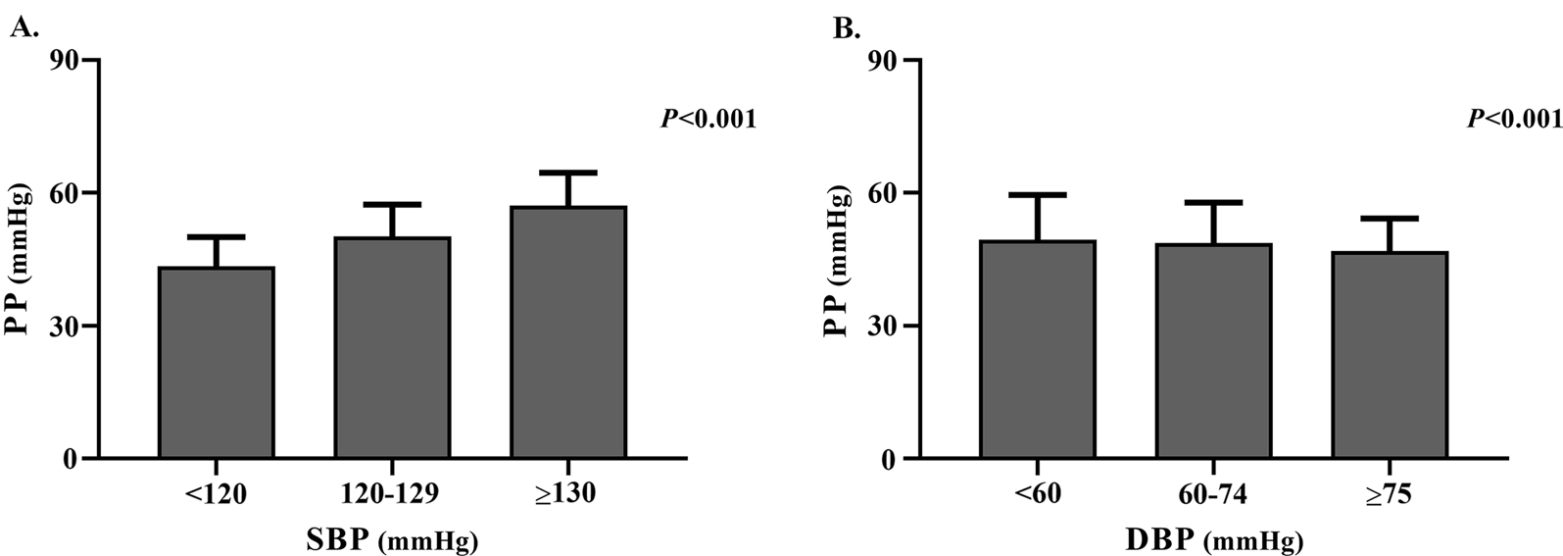
The levels of PP in different BP categories. **A** Comparison of the levels of PP among SBP categories; **B** Comparison of the levels of PP among DBP categories

Higher levels of PP were observed among the participants with higher levels of SBP and lower levels of DBP. (*P* < 0.001) (Fig. 2) Compared with those having eGFR ≥ 90 ml/min/1.73m^2^, significantly higher levels of PP and SBP were observed among participants with lower levels of eGFR. (*P* < 0.001) No significant difference of the levels of DBP was observed among participants with different levels of eGFR. (*P* > 0.05) (Table 1).

### Kidney function and BP components

Results of comparisons of kidney function between participants with different category of BP were presented in Table 2. Altogether 695 participants were recognized as rapid decline of eGFR in the present study. Participants with high PP showed significantly lower levels of eGFR at baseline and at the endpoint. (*P* < 0.001) Much severe annual decline of eGFR and more cases of rapid decline of eGFR were found among participants with high PP. (*P* < 0.05) Compared with participants with low or medium SBP, participants with high SBP showed significantly lower levels of eGFR both at baseline and at the endpoint. (P < 0.001) Participants with low DBP showed lower levels of eGFR at the endpoint and much severe annual decline of eGFR. (P < 0.05) (Table 2).

**Table 2:** Comparison of kidney function among BP categories

Kidney Function	Overall	PP category		*P* value	SBP category			*P* value	DBP category			*P* value
		Normal < 60 mmHg	High ≥ 60 mmHg		Low < 120 mmHg	Medium 120–129 mmHg	High 130–139 mmHg		Low < 60 mmHg	Medium 60–74 mmHg	High 75–89 mmHg	
eGFR in 2011 (ml/min/1.73m^2^, mean ± SD)	96.4 ± 15.6	97.0 ± 15.3	91.0 ± 16.6	< 0.001	97.6 ± 14.9	96.0 ± 16.3	93.8 ± 16.0	< 0.001	95.5 ± 14.8	96.3 ± 15.7	96.8 ± 15.6	0.261
eGFR in 2015 (ml/min/1.73m^2^, mean ± SD)	91.4 ± 17.2	92.2 ± 16.9	84.8 ± 18.8	< 0.001	92.3 ± 17.0	91.5 ± 17.2	88.9 ± 17.8	< 0.001	89.3 ± 18.7	91.0 ± 17.3	92.9 ± 16.6	< 0.001
Annual decline of eGFR (ml/min/1.73m^2^, IQR)	− 0.9 (− 2.8, 0.6)	− 0.9 (− 2.7, 0.6)	− 1.1 (− 3.2, 0.3)	0.013	− 0.9(− 2.9, 0.5)	− 0.9(− 2.5, 0.6)	− 1.0(− 2.9, 0.8)	0.500	− 1.0(− 2.9, 0.5)	− 1.0(2.9, 0.5)	− 0.8(− 2.5, 0.9)	0.011
Rapid decline of eGFR* (n, %)				0.011				0.141				0.236
No	3839(84.7)	3454(85.1)	385(80.7)		2025(84.5)	1024(86.3)	790(83.3)		444(83.6)	2105(84.1)	1290(85.9)	
Yes	695(15.3)	603(14.9)	92(19.3)		373(15.6)	163(13.7)	159(16.8)		87(16.4)	397(15.9)	211(14.1)	

*BP* blood pressure, *PP* pulse pressure, *SBP* systolic blood pressure, *DBP* diastolic blood pressure, *eGFR* estimated glomerular filtration, *SD* standard deviation, *IQR* interquartile range

^*^Rapid decline of eGFR was defined as eGFR decline ≥ 4 ml/min/1.73m^2^/year

### Types of BP component and the rapid decline of eGFR

After fully adjusting for the confounders, high PP (OR = 1.34, 95% CI 1.02–1.75), low SBP (OR = 1.28, 95%CI 1.03–1.59), and high SBP (OR = 1.32, 95% CI 1.02–1.71) were significantly associated with the risk of rapid decline of eGFR, respectively. No significant association was found between DBP category and the risk of rapid decline of eGFR. (*P* > 0.05) (Table 3) Results of unadjusted and partly adjusted models were present in Additional file 1.

**Table 3:** Association between BP category and the risk of rapid decline of eGFR, fully adjusted and age-stratified

Variables	In total (n, %)	Overall		Age 45–54 years		Age 55–64 years		Age ≥ 65 years	
		Adjusted OR	*P* value	Adjusted OR	*P* value	Adjusted OR	*P* value	Adjusted OR	*P* value
PP category									
Normal: < 60 mmHg	4057(89.5)	Ref	Ref	Ref	Ref	Ref	Ref	Ref	Ref
High: ≥ 60 mmHg	477(10.52)	1.34(1.02–1.75)	0.036	1.65(0.83, 3.29)	0.153	1.23(0.79–1.90)	0.364	1.51(1.00–2.27)	0.051
SBP category									
Medium: 120–129 mmHg	1187(26.2)	Ref	Ref	Ref	Ref	Ref	Ref	Ref	Ref
Low: < 120 mmHg	2398(52.9)	1.28(1.03–1.59)	0.025	1.65(1.13–2.43)	0.010	1.05(0.76–1.46)	0.769	1.15(0.73–1.81)	0.550
High: 130–139 mmHg	949(20.9)	1.32(1.02–1.71)	0.032	1.34(0.81–2.22)	0.255	1.41(0.95–2.07)	0.084	1.26(0.78–2.05)	0.345
DBP category									
Low: < 60 mmHg	531(11.7)	Ref	Ref	Ref	Ref	Ref	Ref	Ref	Ref
Medium: 60–74 mmHg	2502(55.2)	0.95(0.72–1.25)	0.706	1.10(0.67–1.82)	0.708	0.99(0.62–1.59)	0.974	0.78(0.48–1.27)	0.321
High: 75–89 mmHg	1501(33.1)	0.83(0.62–1.12)	0.226	0.81(0.48–1.38)	0.446	0.96(0.58–1.59)	0.888	0.77(0.44–1.34)	0.349

Adjusted for age, gender, smoke, drink, BMI, HbA1c, LDL, UA, eGFR at baseline

*BP* blood pressure, *PP* pulse pressure, *SBP* systolic blood pressure, *DBP* diastolic blood pressure, *OR* odds ratio, *Ref.* reference, *BMI* body mass index, *HbA1c* hemoglobin A1c, *LDL* low-density lipoprotein, *UA* uric acid, *eGFR* estimated glomerular filtration rate

Age-stratified analyses were performed in the age groups of 45–54 years, 55–64 years and ≥ 65 years, respectively. Low SBP (OR = 1.65, 95% CI 1.13–2.43) was significantly associated with the increased risk of rapid decline of eGFR among participants aged 45–54 years. No significant association was found between types of BP category and the risk of rapid decline of eGFR among the population aged ≥ 55 years. Results of unadjusted and partly adjusted models were present in Additional file 1.

### BP combination and the rapid decline of eGFR

According to the present results (Table 3), the strategy of combination of PP and SPB was designed to further explore the association between BP components and the risk of rapid decline of eGFR among the middle and old aged general population without pre-existing hypertension. After adjusting for the confounders, reference to the combination of medium SBP and normal PP, the combination of high SBP and high PP (OR = 1.79, 95% CI 1.27–2.54) and the combination of low SBP and high PP OR = 3.07, 95% CI 1.24–7.58) showed significant association with the increased risk of rapid decline of eGFR. (Table 4) Results of unadjusted and partly adjusted models were present in Additional file 2.

**Table 4:** Association between types of BP combination and the risk of rapid decline of eGFR, fully adjusted and age-stratified

Types of BP combination	In total (n, %)	Overall		Age 45–54 years		Age 55–64 years		Age ≥ 65 years	
		OR	*P* value	OR	*P* value	OR	*P* value	OR	*P* value
BP combination									
Medium SBP and Normal PP	1068(23.6)	Ref	Ref	Ref	Ref	Ref	Ref	Ref	Ref
High SBP or High PP	739(16.3)	0.96(0.72–1.29)	0.806	1.09(0.63–1.89)	0.746	1.01(0.66–1.55)	0.965	0.83(0.45–1.51)	0.538
High SBP and High PP	329(7.3)	1.79(1.27–2.54)	0.001	2.65(1.11–6.33)	0.028	1.65(0.96–2.84)	0.069	1.66(0.93–2.95)	0.087
Low SBP and Normal PP	2369(52.3)	1.21(0.97–1.51)	0.094	1.63(1.10–2.40)	0.014	0.97(0.70–1.36)	0.874	1.06(0.65–1.75)	0.796
Low SBP and High PP	29(0.6)	3.07(1.24–7.58)	0.015	14.25(0.92–220.60)	0.057	1.95(0.39–9.86)	0.418	2.90(0.78–10.76)	0.110

Adjusted for age, gender, smoke, drink, BMI, HbA1c, LDL, UA, eGFR at baseline

*BP* blood pressure, *PP* pulse pressure, *SBP* systolic blood pressure, *DBP* diastolic blood pressure, *OR* odds ratio, *Ref.* reference, *BMI* body mass index, *HbA1c* hemoglobin A1c, *LDL* low-density lipoprotein, *UA* uric acid, *eGFR* estimated glomerular filtration rate

Age-stratified analyses showed that the combination of high SBP and high PP (OR = 2.65, 95% CI 1.11–6.33) and the combination of low SBP and normal PP (OR = 1.63, 95% CI 1.10–2.40) were significantly correlated with the increased risk of rapid decline of eGFR among participants aged < 55 years. (Table 4) Results of unadjusted and partly adjusted models were present in Additional file 2.

## Discussion

To find a risk indicator of decline in kidney function among the general population, the present study investigated the association between BP components and the risk of rapid decline of eGFR based on a nationally representative longitudinal study of middle and old aged general population in China. Significant association was observed between PP, SBP and the risk of rapid decline of eGFR among the middle and old aged general population without hypertension, especially among those younger than 55 years. Higher levels of SBP, although lower than 140 mmHg, and excessively low levels of SBP (< 120 mmHg) were correlated to the increased risk of decline in kidney function. PP showed satisfied performance on the risk prediction of decline in kidney function, and the prognostic significance increased with the combination with high SBP. In sum, as an easily-accessible, cost-saving and non-invasive indicator, it is potentially effective to use PP, SBP, and the combination of SBP and PP to monitor the risk of decline in kidney function among the middle and old aged general population.

Increased PP is caused by the elevated SBP and/or decreased DBP and is the results of multiple mechanisms including deficiency of vascular elastin, deterioration of the windkessel function, and decline of left ventricular function. [16, 23] The elevated SBP results in left ventricular hypertrophy leading to the increased afterload whereas the decreased DBP results in the reduced perfusion of coronary artery.[23] Kidney plays essential roles in the homeostasis of hemodynamics.[41] The kidney perfusion pressure precisely responses to the alteration of arterial circulation and BP, and the kidney further regulates the extracellular volume and BP through multiple mechanisms [41]. As to the middle and old aged general population without hypertension, neither the arterial circulation nor the structure of vascular has obvious dysfunction or injury, but the process of ageing gradually impact the vascular physiology and hemodynamics [9]. Alteration of hemodynamics is an important pathogenesis of chronic injury of kidney [41]. Compared with SBP and DBP, the levels of PP much sensitively reflect the changes of systemic hemodynamics, hence showed better prognostic significance. Previously, Rifkin et al. reported a twofold increased risk of rapid decline of kidney function (eGFR loss ≥ 3 ml/min/year) with the PP > 80 mmHg among the elderly (average age: 72.2 years) based on the Cardiovascular Health Study [26]. The present results demonstrated that PP showed satisfactory performance on predicting the risk of decline in kidney function among the Asians and provided the evidence for the prognostic performance of PP among the middle aged population. Although the mechanism of PP and chronic kidney injury remains to be investigated, the present results suggested the potential effectiveness to use PP and the combination of PP and SBP as a surrogate indicator for the risk monitor of CKD in the general population.

The goal of BP on preservation of kidney function among the general population is not clear yet. Generally, the prevention of hypertension (BP < 140/90 mmHg) is considered as the effective approach for CKD prevention among the general population without pre-existing hypertension [8], while few studies further explored the precise BP threshold for kidney function preservation among this population. Results of Systolic Blood Pressure Intervention Trial (SPRINT) strongly suggested the benefits of intensive BP control (SBP < 120 mmHg) on reducing the risk of cardiac events among patients with hypertension and high-risk of CVD [42]. However, the present study found that SBP lower than 120 mmHg showed risk effects on the preservation of kidney function among the middle and old aged general population, especially among those aged 45 to 54 years. It indicated the goals of BP control among the patients with hypertension and among the general population should be different. For the general population without hypertension, intensively low SBP results in the low perfusion of the kidney, which lead to chronic ischemia, hypoxia, and inflammation. Current clinical guidelines of CKD and BP recommended 130/80 mmHg as the goal of BP control among patients with CKD [10, 12]. The present results demonstrated that this threshold was beneficial for kidney function preservation among the general population, but a lower limit of BP was needed. According to the current results, the level of 120–129 mmHg was the satisfactory threshold of SBP on CKD prevention among the middle and old aged general population.

The present study has several limitations. Firstly, information of albuminuria was not available in CHALRS cohort, the association between BP and albuminuria was failed to be analyzed. Secondly, subject to the case number of participants with low SBP and high PP, results of the age-stratified analyses in the combination of low SBP and high PP need to be validated in larger population. Thirdly, the observational nature of our study limited the ability to make causal inference. Fourthly, although ethnic-specific association between genes and BP components were demonstrated in previous studies [30–32, 32], the present study did not investigate the association between genetic factors and the BP components subject to the absence of gene data. Lastly, the possibility of residual confounding exists.

## Conclusion

PP, SBP and the combination of PP and SBP could be surrogate risk indicator for the rapid decline of kidney function among the middle and old aged general population without pre-existing hypertension. Individuals with high PP, excessively low SBP, or elevated SBP near to the threshold of hypertension should be recognized as the high-risk population of rapid decline of kidney function. Risk monitor, CKD screening, and early prevention should be timely initiated among the high-risk population.

## Supplementary Information

**Additional file 1**. Unadjusted and partly adjusted models for the association between BP category and the risk of rapid decline of eGFR, age-stratified.

**Additional file 2**. Unadjusted and partly adjusted models for the association between types of BP combination and the risk of rapid decline of eGFR, age-stratified.

## Data Availability

The data underlying this article are available in the China Health and Retirement Longitudinal Study at http://charls.pku.edu.cn/index/en.html, and can be accessed after application.

## Abbreviations

BP: Blood pressure
BMI: Body mass index
CHARLS: China Health and Retirement Longitudinal Study
CI: Confidence interval
CKD: Chronic kidney disease
CVD: Cardiovascular disease
DBP: Diastolic blood pressure
eGFR: Estimated glomerular filtration rate
HbA1c: Glycosylated hemoglobin
IQR: Interquartile range
LDL: Low-density lipoprotein
OR: Odds ratio
PP: Pulse pressure
SBP: Systolic blood pressure
SCr: Serum creatinine
SD: Standard deviation
UA: Uric acid
