# Supplementary material for: Association between pulse pressure, systolic blood pressure and the risk of rapid decline of kidney function among general population without hypertension: results from the China health and retirement longitudinal study (CHARLS)
Source: J Transl Med. 2021 Dec 20;19:512. doi: 10.1186/s12967-021-03176-8 (PMC8686555; doi:10.1186/s12967-021-03176-8)
Supplement: Supplementary file 1 — Additional file 1. Unadjusted and partly adjusted models for the association between BP category and the risk of rapid decline of eGFR, age-stratified. [file 12967_2021_3176_MOESM1_ESM.docx]

**Additional file 1.** Unadjusted and partly adjusted models for the association between BP category and the risk of rapid decline of eGFR, age-stratified.

| Variables | Unadjusted model | | Model 1 | | Model 2 | |
| --- | --- | --- | --- | --- | --- | --- |
|  | **Crude OR** | ***P* value** | **Adjusted OR** | ***P* value** | **Adjusted OR** | ***P* value** |
| *Overall* |  |  |  |  |  |  |
| PP category |  |  |  |  |  |  |
| Normal:<60mmHg | Ref. | Ref. | Ref. | Ref. | Ref. | Ref. |
| High: ≥60mmHg | 1.37(1.07-1.75) | 0.011 | 1.34(1.04-1.73) | 0.025 | 1.35(1.04-1.75) | 0.024 |
| SBP category |  |  |  |  |  |  |
| Medium: 120-129mmHg | Ref. | Ref. | Ref. | Ref. | Ref. | Ref. |
| Low: <120mmHg | 1.16(0.95-1.41) | 0.150 | 1.22(1.00-1.49) | 0.054 | 1.26(1.02-1.54) | 0.029 |
| High: 130-139mmHg | 1.26(1.00-1.60) | 0.053 | 1.25(0.98-1.59) | 0.067 | 1.26(0.99-1.61) | 0.061 |
| DBP category |  |  |  |  |  |  |
| Low: <60mmHg | Ref. | Ref. | Ref. | Ref. | Ref. | Ref. |
| Medium: 60-74mmHg | 0.96(0.75-1.24) | 0.768 | 0.99(0.76-1.27) | 0.913 | 0.99(0.76-1.29) | 0.935 |
| High: 75-89mmHg | 0.83(0.64-1.10) | 0.193 | 0.83(0.63-1.10) | 0.191 | 0.82(0.62-1.09) | 0.179 |
| *Age 45-54 years* |  |  |  |  |  |  |
| PP category |  |  |  |  |  |  |
| Normal:<60mmHg | Ref. | Ref. | Ref. | Ref. | Ref. | Ref. |
| High: ≥60mmHg | 1.98(1.07-3.68) | 0.030 | 1.84(0.98-3.46) | 0.058 | 2.05(1.08-3.88) | 0.028 |
| SBP category |  |  |  |  |  |  |
| Medium: 120-129mmHg | Ref. | Ref. | Ref. | Ref. | Ref. | Ref. |
| Low: <120mmHg | 1.40(0.99-1.98) | 0.057 | 1.54(1.08-2.18) | 0.016 | 1.64(1.14-2.36) | 0.007 |
| High: 130-139mmHg | 1.28(0.81-2.03) | 0.292 | 1.27(0.80-2.02) | 0.320 | 1.37(0.85-2.21) | 0.197 |
| DBP category |  |  |  |  |  |  |
| Low: <60mmHg | Ref. | Ref. | Ref. | Ref. | Ref. | Ref. |
| Medium: 60-74mmHg | 1.09(0.69-1.72) | 0.707 | 1.12(0.71-1.78) | 0.619 | 1.21(0.75-1.95) | 0.430 |
| High: 75-89mmHg | 0.83(0.51-1.33) | 0.435 | 0.80(0.49-1.29) | 0.357 | 0.82(0.49-1.36) | 0.442 |
| *Age 55-64 years* |  |  |  |  |  |  |
| PP category |  |  |  |  |  |  |
| Normal:<60mmHg | Ref. | Ref. | Ref. | Ref. | Ref. | Ref. |
| High: ≥60mmHg | 1.14(0.75-1.72) | 0.541 | 1.21(0.79-1.83) | 0.380 | 1.25(0.82-1.91) | 0.296 |
| SBP category |  |  |  |  |  |  |
| Medium: 120-129mmHg | Ref. | Ref. | Ref. | Ref. | Ref. | Ref. |
| Low: <120mmHg | 0.99(0.73-1.35) | 0.955 | 1.02(0.75-1.39) | 0.898 | 1.00(0.73-1.37) | 0.989 |
| High: 130-139mmHg | 1.23(0.86-1.77) | 0.258 | 1.26(0.87-1.81) | 0.223 | 1.23(0.85-1.79) | 0.269 |
| DBP category |  |  |  |  |  |  |
| Low: <60mmHg | Ref. | Ref. | Ref. | Ref. | Ref. | Ref. |
| Medium: 60-74mmHg | 1.08(0.69-1.67) | 0.736 | 1.00(0.64-1.57) | 0.984 | 1.01(0.64-1.59) | 0.960 |
| High: 75-89mmHg | 1.08(0.68-1.72) | 0.740 | 0.96(0.60-1.53) | 0.853 | 0.97(0.60-1.57) | 0.895 |
| Age≥65 years |  |  |  |  |  |  |
| PP category |  |  |  |  |  |  |
| Normal:<60mmHg | Ref. | Ref. | Ref. | Ref. | Ref. | Ref. |
| High: ≥60mmHg | 1.15(0.79-1.66) | 0.465 | 1.27(0.87-1.87) | 0.212 | 1.25(0.85-1.86) | 0.259 |
| SBP category |  |  |  |  |  |  |
| Medium: 120-129mmHg | Ref. | Ref. | Ref. | Ref. | Ref. | Ref. |
| Low: <120mmHg | 1.24(0.82-1.86) | 0.310 | 1.16(0.77-1.76) | 0.482 | 1.25(0.81-1.92) | 0.308 |
| High: 130-139mmHg | 1.15(0.74-1.78) | 0.538 | 1.16(0.75-1.81) | 0.507 | 1.24(0.79-1.95) | 0.354 |
| DBP category |  |  |  |  |  |  |
| Low: <60mmHg | Ref. | Ref. | Ref. | Ref. | Ref. | Ref. |
| Medium: 60-74mmHg | 0.81(0.53-1.26) | 0.354 | 0.89(0.57-1.40) | 0.624 | 0.89(0.56-1.41) | 0.619 |
| High: 75-89mmHg | 0.76(0.45-1.26) | 0.280 | 0.80(0.48-1.35) | 0.403 | 0.80(0.47-1.36) | 0.413 |

Model 1: adjusted for age and gender

Model 2: Model 1, smoke, drink, BMI
