# Supplementary material for: Association between pulse pressure, systolic blood pressure and the risk of rapid decline of kidney function among general population without hypertension: results from the China health and retirement longitudinal study (CHARLS)
Source: J Transl Med. 2021 Dec 20;19:512. doi: 10.1186/s12967-021-03176-8 (PMC8686555; doi:10.1186/s12967-021-03176-8)
Supplement: Supplementary file 2 — Additional file 2. Unadjusted and partly adjusted models for the association between types of BP combination and the risk of rapid decline of eGFR, age-stratified. [file 12967_2021_3176_MOESM2_ESM.docx]

**Additional file 2.** Unadjusted and partly adjusted models for the association between types of BP combination and the risk of rapid decline of eGFR, age-stratified.

| Types of BP combination | Unadjusted model | | Model 1 | | Model 2 | |
| --- | --- | --- | --- | --- | --- | --- |
|  | **Crude OR** | ***P* value** | **Adjusted OR** | ***P* value** | **Adjusted OR** | ***P* value** |
| *Overall* |  |  |  |  |  |  |
| BP combination |  |  |  |  |  |  |
| Medium SBP and Normal PP | Ref. | Ref. | Ref. | Ref. | Ref. | Ref. |
| High SBP or High PP | 1.00(0.76-1.31) | 0.993 | 0.97(0.74-1.27) | 0.818 | 0.96(0.72-1.26) | 0.747 |
| High SBP and High PP | 1.67(1.21-2.29) | 0.002 | 1.70(1.23-2.36) | 0.001 | 1.75(1.26-2.44) | 0.001 |
| Low SBP and Normal PP | 1.12(0.91-1.38) | 0.268 | 1.17(0.95-1.45) | 0.132 | 1.20(0.97-1.49) | 0.087 |
| Low SBP and High PP | 2.35(1.02-5.40) | 0.044 | 2.41(1.03-5.64) | 0.042 | 2.45(1.05-5.74) | 0.039 |
| *Age 45-54 years* |  |  |  |  |  |  |
| BP combination |  |  |  |  |  |  |
| Medium SBP and Normal PP | Ref. | Ref. | Ref. | Ref. | Ref. | Ref. |
| High SBP or High PP | 1.10(0.67-1.81) | 0.701 | 1.09(0.66-1.79) | 0.747 | 1.17(0.70-1.95) | 0.561 |
| High SBP and High PP | 2.61(1.20-5.68) | 0.016 | 2.59(1.17-5.74) | 0.019 | 3.05(1.36-6.85) | 0.007 |
| Low SBP and Normal PP | 1.40(0.98-1.99) | 0.062 | 1.53(1.07-2.19) | 0.019 | 1.64(1.14-2.38) | 0.008 |
| Low SBP and High PP | 7.83(1.08-56.90) | 0.042 | 6.57(0.87-49.64) | 0.068 | 8.67(1.08-69.51) | 0.042 |
| *Age 55-64 years* |  |  |  |  |  |  |
| BP combination |  |  |  |  |  |  |
| Medium SBP and Normal PP | Ref. | Ref. | Ref. | Ref. | Ref. | Ref. |
| High SBP or High PP | 1.02(0.68-1.52) | 0.939 | 1.01(0.67-1.53) | 0.946 | 0.97(0.64-1.47) | 0.893 |
| High SBP and High PP | 1.34(0.80-2.23) | 0.269 | 1.46(0.87-2.45) | 0.156 | 1.51(0.89-2.53) | 0.128 |
| Low SBP and Normal PP | 0.94(0.69-1.29) | 0.719 | 0.97(0.71-1.34) | 0.872 | 0.95(0.69-1.32) | 0.768 |
| Low SBP and High PP | 1.31(0.28-6.20) | 0.733 | 1.48(0.31-7.11) | 0.627 | 1.48(0.31-7.12) | 0.628 |
| *Age ≥65 years* |  |  |  |  |  |  |
| BP combination |  |  |  |  |  |  |
| Medium SBP and Normal PP | Ref. | Ref. | Ref. | Ref. | Ref. | Ref. |
| High SBP or High PP | 0.78(0.45-1.33) | 0.359 | 0.75(0.43-1.30) | 0.306 | 0.76(0.43-1.33) | 0.337 |
| High SBP and High PP | 1.32(0.78-2.23) | 0.295 | 1.41(0.83-2.40) | 0.202 | 1.49(0.86-2.56) | 0.154 |
| Low SBP and Normal PP | 1.13(0.73-1.76) | 0.582 | 1.05(0.67-1.65) | 0.821 | 1.11(0.70-1.76) | 0.661 |
| Low SBP and High PP | 1.81(0.54-6.09) | 0.341 | 2.02(0.58-6.98) | 0.266 | 2.09(0.59-7.36) | 0.251 |

Model 1: adjusted for age and gender

Model 2: Model 1, smoke, drink, BMI
